# Atrial fibrillation secondary to diffuse large B-cell lymphoma responding to corticosteroid treatment: a case report

**DOI:** 10.1093/ehjcr/ytag371

**Published:** 2026-06-19

**Authors:** Luca Martini, Iacopo Fabiani, Chrysanthos Grigoratos, Claudio Passino

**Affiliations:** Division of Cardiology, Fondazione Toscana Gabriele Monasterio, Via Moruzzi 1, Pisa 56124, Italy; Department of Heart, Chest, and Vessels, Cardiology Unit, Azienda Ospedaliera Universitaria Senese, viale Bracci 11, 53100 Siena, Italy; Division of Cardiology, Fondazione Toscana Gabriele Monasterio, Via Moruzzi 1, Pisa 56124, Italy; Division of Cardiology, Fondazione Toscana Gabriele Monasterio, Via Moruzzi 1, Pisa 56124, Italy; Division of Cardiology, Fondazione Toscana Gabriele Monasterio, Via Moruzzi 1, Pisa 56124, Italy; Health Science Interdisciplinary Centre, Scuola Superiore Sant’Anna, via Moruzzi 1, 56124 Pisa, Italy

**Keywords:** Atrial fibrillation, Lymphoma, Corticosteroid

Learning points

- Inflammatory mechanisms and extrinsic atrial compression should be considered in cases of new-onset atrial fibrillation without traditional cardiovascular risk factors.
- Corticosteroid therapy may lead to rapid rhythm normalization in selected patients with lymphoma-associated atrial arrhythmias.

## Introduction

New-onset atrial fibrillation (AF) is frequently multifactorial and may be related to advanced age, structural heart disease, acute systemic illness, inflammation, metabolic derangements, or drug exposure. In oncologic patients, additional mechanisms such as tumour infiltration, extrinsic compression, or paraneoplastic inflammation may contribute.^1^

AF is also a recognized comorbidity in patients with non-Hodgkin lymphoma (NHL), likely influenced by the immunosuppressive effects of treatment regimens such as corticosteroids, chemotherapy, and monoclonal antibodies.^2,3^ In some cases, however, new-onset AF may represent the initial clinical manifestation of an underlying lymphoma, even in the absence of typical systemic symptoms.^4^ Recognizing this atypical presentation is essential, as early arrhythmia onset may reflect occult cardiac infiltration or compression.

We describe the case of a patient with AF secondary to left atrial (LA) compression by a diffuse large B-cell lymphoma (DLBCL), in whom sinus rhythm was restored following the initiation of corticosteroid therapy alone.

## Summary figure

**Figure:**
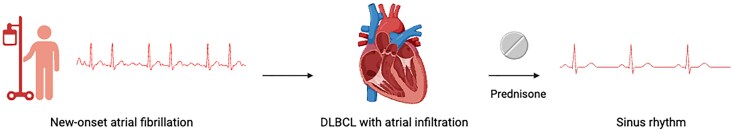


## Case report

A 76-year-old Caucasian male, a former smoker (15 pack-years), presented in January 2025 to the pre-admission clinic of a general hospital for a routine preoperative electrocardiogram (ECG) prior to surgery for nephrolithiasis. The ECG revealed a new diagnosis of atrial fibrillation. The patient had no prior history of atrial arrhythmias, heart failure, coronary artery disease, diabetes mellitus, or thyroid dysfunction. Apart from nephrolithiasis, no relevant comorbidities were reported. At presentation, the patient had a CHA_2_DS_2_-VA score of 2 (age). Transthoracic echocardiography showed preserved left ventricular ejection fraction, normal left ventricular dimensions, and no criteria for heart failure with preserved ejection fraction (HFpEF).

Anticoagulation with low-molecular-weight heparin (LMWH) was initiated until the end of February. Subsequent serial Holter ECGs between January and April documented paroxysmal AF. However, oral anticoagulation was not initiated. The reason why the patient’s GP did not initiate anticoagulation during this period is unknown.

In May 2025, the patient presented to the emergency department with palpitations; the ECG confirmed AF with a rapid ventricular response. Laboratory testing revealed elevated NT-proBNP (4400 ng/L), high-sensitivity troponin T (17 ng/L), C-reactive protein (3.35 mg/dL), and a serum creatinine of 0.94 mg/dL. Complete blood count was within normal limits, with no anaemia, leucocytosis, or thrombocytopenia. Transoesophageal echocardiography, performed to exclude left atrium appendage thrombus in the context of a potential rhythm-control strategy, excluded intracavitary masses but demonstrated marked circumferential hypertrophy of the LA wall (10–11 mm). Chest X-ray showed signs of hilar congestion, and abdominal ultrasonography revealed bilateral pleural effusions.

**Figure 1:**
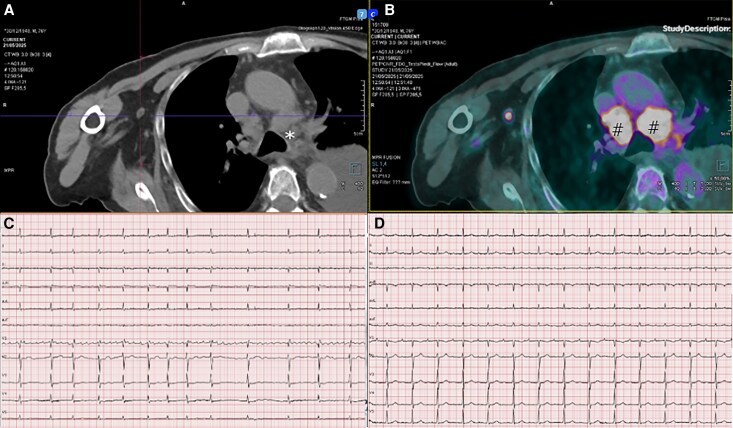
*A*) Chest computed tomography showing cardiac infiltration (*). *B*) Positron emission tomography evidencing increased metabolic activity atria level (#). *C*) ECG prior chemotherapy with atrial fibrillation and *D*) after therapy with sinus rhythm and atrio-ventricular block.

The patient was admitted to a tertiary referral hospital, where a chest computed tomography (CT) scan identified an anterior mediastinal mass (*Figure 1*). A subsequent positron emission tomography-CT (PET-CT) demonstrated increased metabolic activity in the mediastinal mass (SUV max 28.8), a peri-aortic abdominal mass (SUV max 30.8), and several lymph nodes (para-aortic SUV max 6.6; right axillary SUV max 10.0; retroclavicular SUV max 7.9; left axillary SUV max 6.2) (*Figure 1*). Biopsies of both abdominal and thoracic masses confirmed the diagnosis of DLBCL.

During this diagnostic workup, the patient remained in persistent AF. Following the haematological consultation, corticosteroid therapy with prednisone was initiated. Besides serial Holter ECG, serial transthoracic echocardiographs were performed to monitor the evolution of tumour-related infiltration or compression. A low-dose beta-blocker was used transiently for ventricular rate control. No antiarrhythmic drugs were initiated prior to corticosteroid therapy. Given the paroxysmal nature of AF and the absence of significant symptoms prior to oncologic diagnosis, catheter ablation was not considered at this stage. Rhythm control strategies were deferred pending oncologic stabilization. Oral anticoagulation with edoxaban was started.

Within 2 days, ECG demonstrated conversion to sinus rhythm, accompanied by a first-degree atrioventricular block, which was transient and resolved spontaneously during subsequent monitoring. After restoration of sinus rhythm, the patient remained under close rhythm surveillance. Long-term rhythm control strategies were deferred, given the absence of AF recurrence and the priority of oncologic treatment. The patient received the first two cycles of chemotherapy and monoclonal antibody therapy while hospitalized, with no recurrence of AF during monitoring. At the last follow-up visit, the patient remained asymptomatic from a cardiovascular standpoint, in sinus rhythm, with no documented AF recurrence. Oral anticoagulation with edoxaban was maintained.

## Discussion

This case highlights a rare but clinically significant presentation of AF secondary to LA compression from a DLBCL. Cardiac involvement by lymphomas is uncommon, and when present, often remains clinically silent until the disease reaches an advanced stage.^5–8^ The mechanism by which lymphomas induce arrhythmias is multifactorial and may involve direct myocardial infiltration, compression of cardiac chambers or conduction pathways, inflammatory mediators, or metabolic disturbances associated with the neoplastic process.^6,7^

In this patient, transthoracic and transoesophageal echocardiography excluded the presence of intracavitary masses but revealed abnormal left atrial wall thickening. This finding, in conjunction with imaging evidence of an adjacent mediastinal mass and elevated metabolic activity on PET-CT, supported the hypothesis of extrinsic compression or infiltration of the atrial myocardium as a potential trigger for AF. Notably, the arrhythmia resolved rapidly following the initiation of corticosteroid therapy, before the administration of definitive chemoimmunotherapy, suggesting a strong inflammatory or mass effect component in the pathogenesis of AF in this case.^8^

The reversion to sinus rhythm after only 2 days of steroid therapy underscores the potential role of inflammation and oedema in atrial arrhythmogenesis in the setting of cardiac lymphoma. Corticosteroids, known for their potent anti-inflammatory effects, may have reduced peritumoral oedema and myocardial irritation, thereby restoring electrical stability. This observation is consistent with previous case reports describing rhythm normalization after anti-lymphoma treatment, including corticosteroids.^2,8^

Although the temporal association between lymphoma diagnosis, atrial involvement on imaging, and rapid rhythm normalization after corticosteroid therapy suggests a possible causal relationship, a definitive cause-and-effect link cannot be established. The patient may have had an undiagnosed paroxysmal AF that progressed independently of the tumour. Therefore, this case highlights a plausible association rather than definitive causality.

Importantly, the patient did not experience recurrence of AF during hospitalization or initiation of systemic therapy, suggesting that early intervention may have not only diagnostic but also therapeutic value in cardio-oncology. The transient atrioventricular conduction delay noted on ECG after cardioversion also raises the possibility of lymphoma-related involvement of the conduction system, which resolved with treatment.

This case also carries important clinical implications for AF management in the context of newly diagnosed lymphoma. Decisions regarding rate control must account for potential conduction system involvement or haemodynamic compromise due to mass effect. Rhythm control strategies—including cardioversion or antiarrhythmic therapy—should be approached cautiously until infiltrative disease is excluded or stabilized. Anticoagulation strategies must balance thromboembolic risk with the elevated bleeding risk associated with invasive diagnostic procedures and chemotherapy. A multidisciplinary cardio-oncology approach is therefore essential to optimize timing and selection of therapies.

AF in patients with lymphoma has relevant clinical implications, including increased thromboembolic risk, challenges in anticoagulation management, and potential interactions between antiarrhythmic and oncologic therapies.^7,8^ Moreover, AF may reflect advanced disease burden or cardiac involvement and has been associated with worse short-term outcomes in oncologic populations.^5–8^ These considerations underscore the importance of a multidisciplinary cardio-oncology approach.

Furthermore, this case illustrates the importance of considering extracardiac causes, including malignancy, in the differential diagnosis of new-onset or persistent AF, particularly in the absence of traditional risk factors. In patients with systemic symptoms or abnormal cardiac imaging, prompt integration of oncologic evaluation and advanced imaging modalities, such as PET-CT, may facilitate early diagnosis and tailored management. Finally, this case emphasizes the therapeutic potential of corticosteroids in selected patients with lymphoma-associated arrhythmias, even prior to definitive oncologic treatment.

## Conclusions

AF may represent an early sign of cardiac involvement in DLBCL. In this case, sinus rhythm was rapidly restored with corticosteroid therapy alone, suggesting a reversible, inflammation-driven mechanism. Clinicians should consider malignancy in the differential diagnosis of new-onset AF, especially when imaging reveals atypical cardiac findings. Early anti-inflammatory treatment may support both arrhythmia management and diagnostic workup.

## Data Availability

The data underlying this article are available within the article and its supplementary material. Further details are available from the corresponding author upon reasonable request.

## Lead author biography

Physician.Cardiologist.Post-doc course 2nd level Master programme in advanced echocardiography in clinical practice.Post-doc course 2nd level Master programme in clinical competence in heart failure.Post-doc course 2nd level Master programme in cardiac intensive care unit.HFA certified heart failure specialist.

## Notes

**Consent:** Written informed consent was obtained from the patient for the publication of this case report and any accompanying images. A copy of the written consent is available for review by the Editor of this journal, in accordance with COPE guidelines.
